# Editorial: 15 years of frontiers in human neuroscience: new insights in cognitive neuroscience

**DOI:** 10.3389/fnhum.2025.1601465

**Published:** 2025-06-12

**Authors:** Elias Ebrahimzadeh, Hamid Soltanian-Zadeh

**Affiliations:** ^1^Control and Intelligent Processing Center of Excellence (CIPCE), School of Electrical and Computer Engineering, College of Engineering, University of Tehran, Tehran, Iran; ^2^School of Cognitive Sciences, Institute for Research in Fundamental Sciences (IPM), Tehran, Iran

**Keywords:** cognitive neuroscience, reinforcement learning, fNIRS, EEG, fMRI, neural dynamics

## Introduction

In recent years, scientists have developed novel techniques and accomplished remarkable achievements, resulting in substantial advancements within the rapidly expanding field of Human Neuroscience. In this context, engineering methodologies applied to cognitive neuroscience have consistently played a crucial role and have garnered widespread attention from researchers worldwide. A Research Topic centered on new perspectives in cognitive neuroscience offers an in-depth exploration of the latest advancements and developments in the field. It that allows researchers to share their findings and ideas, fostering collaboration and knowledge exchange. Such a Research Topic enables a detailed assessment of the field's current status while providing a valuable resource for the scientific community and beyond. Furthermore, it plays a crucial role in raising awareness about the importance of advancing cognitive neuroscience, promoting further research, and ultimately contributing to more precise diagnoses and effective treatments for individuals facing challenges in this area.

This Research Topic aims to explore new insights, novel developments, current challenges, recent discoveries, latest advancements, and future directions in the field of cognitive neuroscience. It invited concise and forward-thinking contributions from researchers, highlighting the state of the art in the field. These contributions outline significant recent achievements, as well as the critical steps needed to drive the field forward. Authors were encouraged to pinpoint the most pressing challenges within specific subfields and propose innovative strategies in cognitive neuroscience to overcome these challenges.

While the articles included in this Research Topic do not primarily focus on reviewing the progress made over the past decade, they highlight recent advances, novel developments, current challenges, and emerging perspectives in cognitive neuroscience. This Research Topic will inspire, inform, and provide direction and guidance to researchers in the field. We aim to improve the understanding of the relation between cognitive processes and resting state networks, the dynamics of cognitive processes, and applications of machine learning methodologies on biomedical signals and images, and the relationship between findings. Methods and applications in cognitive neuroscience using biomedical signal/image processing aim to highlight the latest experimental techniques and methods for investigating the fundamental questions regarding the mental processes involved in cognition.

## Research Topic coverage

This Research Topic includes four original research articles and two review articles. The accepted papers cover new insights, novel developments, current challenges, latest discoveries, recent advances, and future perspectives in the field of cognitive neuroscience.

Both adults and children learn through feedback to associate environmental events and choices with reward, a process known as reinforcement learning (RL). However, tasks to assess RL-related neurocognitive processes in children have been limited. The paper entitled “*Electrical brain activations in preadolescents during a probabilistic reward-learning task reflect cognitive processes and behavior strategies*” by Chung et al. validated a child version of the Probabilistic Reward Learning task in preadolescents (8–12 years) while recording event-related potentials (ERPs). Behaviorally, as expected, preadolescents could learn stimulus–reward outcome associations, but with varying performance levels. Poor learners showed greater RewP amplitudes compared to good learners. These findings provide novel insights into the neural processes underlying RL in preadolescents.

Zahar et al. have conducted a systematic review investigating the acute cognitive effects of dietary compounds using fNIRS. They have summarized methodological limitations and perspectives for research targeting healthy adults. This review proposes recommendations to enhance current methodologies in the research field, focusing on key aspects of the data collection phase, including research design, experimental paradigms, and participant demographics, and their integration into the analysis phase. Ultimately, it seeks to advance our understanding of the effects of bioactive compounds on cognitive functions to contribute to the development of targeted nutritional interventions for improved brain health.

Nomura et al. have used fMRI to show that the supplementary motor area is deactivated during mental rotation tasks with biomechanical constraints. They investigated cerebral activation while each participant decided whether a hand-palm image, rotated by 0°, 90°, 180°, and 270°, was a right or left hand. A significant negative correlation between the angle and brain activity was observed in the right and left supplementary motor area (SMA) and right posterior anterior cingulate gyrus. This study provided novel findings regarding the neurophysiological mechanisms of motor imagery.

The paper entitled “*Neural dynamics of delayed feedback in robot teleoperation: insights from fNIRS analysis*” by Zhou et al. addresses a gap in the literature by leveraging fNIRS to examine the neurofunctional implications of simulated haptic feedback on cognitive activity and motor coordination under delayed conditions. The fNIRS data provided a detailed assessment of cerebral activity, particularly in the regions of interest (ROIs) implicated in time perception and the execution of precise movements. Their results reveal that the anchoring condition, which provided immediate simulated haptic feedback alongside a delayed visual cue, significantly optimized neural functions related to time perception and motor coordination. This condition also improved motor performance compared to the asynchronous condition, where visual and haptic feedback were misaligned.

Minamoto et al. used a self-referential task to investigate the effect of normobaric hypoxia on the self-referential process. They measured brain activity during the task using fNIRS and performed conventional univariate analysis with the General Linear Model (GLM) and homologous cortical functional connectivity analysis. The results revealed that normobaric hypoxia impaired the recognition of adjectives in the other-reference condition but not in the self-reference condition. The GLM analysis did not detect differences in brain activity between the self- and other-reference conditions, suggesting that it may not be suitable for examining the neural correlates of these conditions.

Azadi Moghadam and Maleki conducted a systematic review and meta-analysis on fatigue indices and fatigue caused by stimulation paradigms. Attractiveness and variation are the most effective ways to reduce brain computer interface (BCI) fatigue. Therefore, zoom motion, Newton's ring motion, and cue patterns are effective in reducing fatigue. While the color of the cue could effectively reduce fatigue, its shape and background did not have an impact. The outcomes of this study can be used to design more appropriate stimulation protocols that cause less fatigue. Moreover, the level of fatigue can be quantitatively assessed using indicators without relying on participants' self-reports.

All of the articles present important ideas for identifying the brain regions and neural networks involved in different cognitive tasks or conditions. The editors are pleased to present this Research Topic of articles to the field of cognitive neuroscience and related scientific communities. They hope that this Research Topic is found interesting by the readers of the articles and the researchers working in the related fields of medicine, biomedical engineering, and neuroscience.

